# PILOT OUTCOMES OF A FALL PREVENTION PROGRAM “LIVE-LIFE” FOR COMMUNITY-DWELLING OLDER ADULTS IN CHINA

**DOI:** 10.1093/geroni/igae098.0364

**Published:** 2024-12-31

**Authors:** Yuqian Luo, Cen Mo, Wenting Peng, Sarah Szanton, Minhui Liu

**Affiliations:** Hong Kong Polytechnic University, Hong Kong, Hong Kong, China (People’s Republic); Central South University, Changsha, Hunan, China (People’s Republic); University of Washington, Seattle, Washington, United States; Johns Hopkins University, Baltimore, Maryland, United States; Ningxia Medical University, Yinchuan, Ningxia, China

## Abstract

More than 70 million older adults fall at least once each year in China, which may lead to disability, fear of falling, social isolation and even death. We conducted a pilot randomized controlled trial to evaluate the preliminary effects of the multi-component LIVE-LiFE program on health-related outcomes among community-dwelling older adults in China. Thirty-Eight participants, average 70.32 years old, 76.3% of female, were randomized to either the intervention group (n=20) or the control group (n=18) for 11 weeks. In the intervention group, participants received the LIVE-LiFE program (individualized strength and balance training, vision screening, medication review, and home hazards assessment and modification), whereas the control group received equal amounts of health education on fall prevention. To compare the differences of pre-post scores between two groups, we employed two independent sample t-test and independent sample Wilcoxon signed-rank test. Results indicated significant differences and moderate effect sizes on 3-meter walking speed (P< 0.001, d =-0.603) and home hazards (P=0.043, d=-0.504). The paired t-test and paired Wilcoxon signed-rank test were used to examine the paired LiFE assessment data of the intervention group. Significant improvements were observed on the total scores (P< 0.001, d=1.260), balance function dimension scores (P< 0.001, d=0.962), and strength function dimension scores (P=0.002, d=0.776). Our study showed positive preliminary effects of the LIVE-LiFE program on improving both personal function and environmental safety. Future research should consider both internal and external factors to prevent falls among community-dwelling older adults with larger sample sizes and longer follow-up.

